# Responsiveness of genes to manipulation of transcription factors in ES cells is associated with histone modifications and tissue specificity

**DOI:** 10.1186/1471-2164-12-102

**Published:** 2011-02-09

**Authors:** Alexei A Sharov, Akira Nishiyama, Yulan Piao, Lina S Correa-Cerro, Tomokazu Amano, Marshall Thomas, Samir Mehta, Minoru SH Ko

**Affiliations:** 1 Developmental Genomics and Aging Section, Laboratory of Genetics, National Institute on Aging, NIH, Baltimore, MD 21224, USA; 2 Department of Immunology, Graduate School of Medicine and Faculty of Medicine, Yokohama City University, Yokohama, Kanagawa 236-0004, Japan

## Abstract

**Background:**

In addition to determining static states of gene expression (high vs. low), it is important to characterize their dynamic status. For example, genes with H3K27me3 chromatin marks are not only suppressed but also poised for activation. However, the responsiveness of genes to perturbations has never been studied systematically. To distinguish gene responses to specific factors from responsiveness in general, it is necessary to analyze gene expression profiles of cells responding to a large variety of disturbances, and such databases did not exist before.

**Results:**

We estimated the responsiveness of all genes in mouse ES cells using our recently published database on expression change after controlled induction of 53 transcription factors (TFs) and other genes. Responsive genes (*N *= 4746), which were readily upregulated or downregulated depending on the kind of perturbation, mostly have regulatory functions and a propensity to become tissue-specific upon differentiation. Tissue-specific expression was evaluated on the basis of published (GNF) and our new data for 15 organs and tissues. Non-responsive genes (*N *= 9562), which did not change their expression much following any perturbation, were enriched in housekeeping functions. We found that TF-responsiveness in ES cells is the best predictor known for tissue-specificity in gene expression. Among genes with CpG islands, high responsiveness is associated with H3K27me3 chromatin marks, and low responsiveness is associated with H3K36me3 chromatin, stronger tri-methylation of H3K4, binding of E2F1, and GABP binding motifs in promoters.

**Conclusions:**

We thus propose the responsiveness of expression to perturbations as a new way to define the dynamic status of genes, which brings new insights into mechanisms of regulation of gene expression and tissue specificity.

## Background

Gene expression is regulated by the interplay of various kinds of factors including transcription factors (TFs) that bind to DNAs in a sequence-specific manner, the chromatin structure [1-4], and the association of genes with the nuclear lamina/matrix [5,6]. Many TFs directly switch gene expression on or off, whereas others factors may serve as constraints (e.g., by controlling the access of TFs to DNA). Thus, in addition to identifying static states of gene expression (e.g., high vs. low) it is important to characterize the dynamic status, which is the capacity to modify the level of expression. For example, genes with H3K27me3 chromatin marks at promoters are not only suppressed (static state) but also poised for activation (dynamic state) [7]. Responsiveness of genes to perturbations is a dynamic property that was never studied systematically because such studies require the analysis of expression profiles of cells responding to a large variety of disturbances, and such databases did not exist before. The only comparable study used indiscriminately all data for a specific human array platform in the Gene Expression Omnibus (GEO) database, which included both perturbations of the same cell type as well as differences between various cell types, tissues, and organs [8]. Thus, it did not distinguish between responsiveness of genes and their tissue-specific expression.

This paper examines the responsiveness of all genes in mouse ES cells estimated using our recently published database on gene expression changes after controlled induction of 53 transcription factors (TFs) and other genes [9]. In this experiment, we established mouse ES cell lines in which individual transgenic TFs were induced by the removal of doxycycline. Each manipulated TF modifies the expression of many downstream target genes, including other TFs, which can in turn activate or repress genes even farther downstream. Thus, we can quantify responsiveness of not only direct targets of manipulated TFs, but also indirect (i.e., secondary, tertiary) target genes. We show that responsive genes mostly have regulatory functions and a propensity to become tissue-specific upon differentiation, whereas many non-responsive genes have housekeeping functions. To examine the relationship between the responsiveness and tissue-specificity of gene expression, we performed whole-genome expression profiling of 15 mouse adult organs/tissues. Responsiveness of genes in undifferentiated ES cells appears to be a better predictor for tissue-specific gene expression than other known markers (presence of a CpG island and TATA box). Among genes with CpG islands, responsiveness is strongly associated with their epigenetic marks (e.g., histone modifications such as H3K27me3 and H3K36me3), as well as with binding of certain TFs in promoters. These results suggest that TF-responsiveness can be used as a novel indicator of the dynamic status of genes.

## Results

### Definition of TF-responsiveness

We define TF-responsiveness as a gene's readiness for expression change, irrespective of direction (up or down), upon an induced change in the abundance of various TFs. Some genes may change their expression following the manipulation of a wide range of TFs, whereas others may react specifically to one or a few TFs. It is thus important to assess TF-responsiveness based on the data from a wide range of TF manipulations. To differentiate between upregulation and downregulation of genes, we use three indicators of TF-responsiveness for each gene: maximum logratio for upregulation, *U*_*i*_; downregulation, *D*_*i*_; and the average for both up- and downregulation, *B*_*i*_:

(1) $$U_{i}=\underset{j}{\max}(x_{ij});\;D_{i}=\underset{j}{\max}(-x_{ij});\;B_{i}=\text{average(}U_{i},D_{i}\text{)};$$

where *x*_*ij *_is the logratio of expression change of *i*-th gene after perturbation *j*. Term "responsiveness" is similar to "sensitivity", however the latter term is traditionally applied to a single type of perturbation (as in "sensitivity analysis"). Thus, here we use the term "responsiveness" to describe the combined sensitivity of gene expression to various perturbations.

### Data sets used in the study

**Figure 1:**
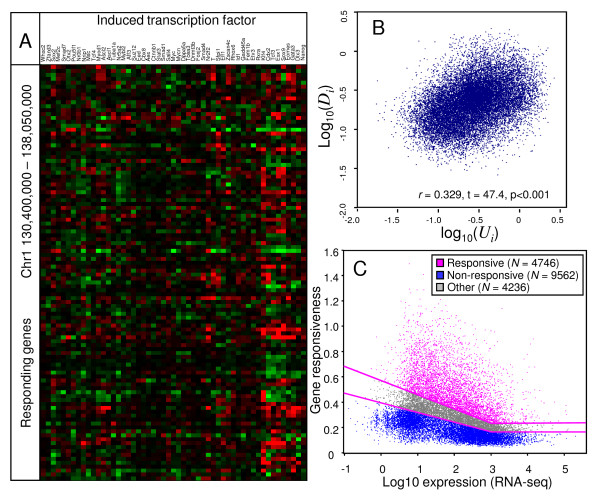
**TF-responsiveness of genes in ES cells**. (A) A heatmap of gene response to the induction of 50 transcription factors and 3 other genes in ES cells; responding genes are plotted in the order of their genome locations in chr1:130,400,000-138,050,000, as listed in Additional File 2. (B) Correlation between log-transformed TF-responsiveness of genes for upregulation and downregulation. (C) TF responsiveness plotted against log-expression of genes estimated from published RNA-seq data [19]; groups of responsive, non-responsive, and borderline genes are shown by color.

A list of gene expression data sets used in this study is given in Additional File 1. To analyze TF-responsiveness of genes in ES cells, we used the following two data sets. First, the "NIA ES bank, 53 genes" data includes gene expression profiles of ES cells 2 days after the induction of each of 53 genes (50 TFs and 3 other genes) [9]. Figure 1A shows a typical response of genes (*x*_*ij*_) located sequentially in a 7.65 Mb window on Chromosome 1. Second, the "NIA Other ES perturbations" data include time-courses expression profiles 1-3 days after downregulation of the *Pou5f1 *or *Sox2 *genes [10-12], and expression profiles of ES cells 2 days after Leukemia Inhibitory Factor (LIF) removal, addition of retinoic acid (RA) [13], or addition of inhibitors of FGFR (PD173074), MEK (PD98059), and GSK (BIO) signaling pathways. The expression profiles of ES cells after treating them with inhibitors of FGFR, MEK, and GSK for 2 days were newly generated for this work and submitted to the public database (GEO accession number GSE19814). The results showed that all three inhibitors downregulated *Rxrg *and *Wt1 *and upregulated *Gbx2 *and *Plagl1 *(*Zac1*). We also observed inhibitor-specific effects: FGFR inhibitor caused upregulation of *Gata3 *and *Gata2 *and downregulation of *Myc*; MEK inhibitor caused upregulation of *Gbp3 *and downregulation of *Erg1 *and *Nr4a3*; and GSK inhibitor caused upregulation of *T*, *Nkx1-2*, *Msx1*, and *Evx1 *and downregulation of *Tcfec *and *Rfx4*.

To analyze the tissue specificity of gene expression, we used two datasets of expression profiles. First, the "NIA Differentiated cells/tissues" set includes expression profiles of 15 mouse adult organs and tissues (new data: GEO accession number GSE19806), as well as published data on gene expression in trophectoderm stem (TS) cells, neural stem (NS) cells, placenta, several lines of fibroblasts, and newborn mice [10,14]. The "NIA Differentiated cells/tissues" microarray data were newly generated for this work and submitted to the public database (GEO accession number GSE19806). Second, the "GNF Mouse tissues" set includes expression profiles of 51 tissues from the mouse Gene Atlas V2 [15] after excluding expression profiles of oocytes, early embryos, and gonads, as they may include undifferentiated cells.

We also used TF binding data, which was inferred from published ChIP-seq data [9,16,17] as well as data on whole-genome chromatin modifications for H3K4me3, H3K27me3, H3K36me3 [7], and H3K9me3 [18]. Relative expression of genes in ES cells was estimated using RNA-seq data [19] and transcript coordinates from the NIA Mouse Gene Index [20]. See the Methods section for further detail.

### Estimating the TF-responsiveness

Indicators of TF-responsiveness (*U*_*i*_, *D*_*i *_and *B*_*i*_) were estimated for 18,544 non-redundant genes based on the "NIA ES bank, 53 genes" data set (Additional Files 2, 3). Although the microarray platform we used represented 25,030 non-redundant genes, we analyzed only those genes (*N *= 18,544) whose expression was determined with sufficient accuracy and whose transcription start sites (TSS) were known (see Methods for further detail). TF-responsiveness for upregulation (*U*_*i*_) and downregulation (*D*_*i*_) are positively correlated (*r *= 0.329 in the log-scale), which indicates that genes upregulated by overexpression of some TFs tend to be downregulated by overexpression of other TFs (Figure 1B). A heatmap also showed that many genes that were strongly upregulated following one perturbation were also strongly downregulated by another kind of perturbation (Figure 1A). The positive correlation between *U*_*i *_and *D*_*i *_was the basis for considering TF-responsiveness as a general dynamic state of a gene that can be applied to both upregulation and downregulation of its expression. To check if measurements of TF-responsiveness were reproducible, we analyzed an independent data set "NIA Other Perturbations." Estimates of TF-responsiveness, *B*_*i*_, from both data sets showed strong correlation (*r *= 0.614, in the log-scale), indicating a high level of reproducibility and independence from the perturbation type (Additional Files 4, 5).

As the spectrum for TF-responsiveness of genes was continuous, we split all the genes examined for expression profiling into three parts: responsive (top 25% of genes), borderline, and non-responsive (bottom 50% of genes) genes (Figure 1C). Because genes with low expression generally had higher levels of TF-responsiveness than highly-expressed genes, we used floating thresholds to separate these groups of genes (Figure 1C) (see details in Methods). All responsive genes had statistically significant responses to manipulation of TFs, based on ANOVA (FDR < 0.05). Further analysis is focused on the comparison of two extreme groups of genes: responsive (*N *= 4746) and non-responsive (*N *= 9562).

Because there is a possibility that genes with high expression show weak response to perturbations simply due to saturation or miscalibration of microarray signals, we tested the sensitivity of the microarray by serial dilution of mRNA [14]. Our results showed that saturation was detected only in 0.2% of genes, and microarray signals were well-calibrated in the full dynamic range of gene expression levels. The difference in TF-responsiveness among genes cannot be attributed to differential mRNA stability because groups of responsive and non-responsive genes had similar distributions of mRNA decay rates (Additional File 6). Data on mRNA degradation was taken from our database (http://lgsun.grc.nia.nih.gov/mRNA) [14]. The mean mRNA decay rate of responsive genes (0.1307 hr^-1^) was only 15.4% higher than that of non-responsive genes (0.1133 hr^-1^); this difference is too small to explain a 3-fold gap between averages of TF-responsiveness in these groups of genes (0.5224 vs. 0.1745).

### Functional annotation of responsive and non-responsive genes

To characterize responsive and non-responsive genes, we first analyzed their possible functions by examining Gene Ontology (GO) classifications. Overrepresented GO terms for responsive genes included various kinds of regulatory functions (e.g. "organ development", "transcription factor", "nervous system development", and "cell motility") (Table 1). The full list of overrepresented GO terms with statistical analysis can be found in Additional File 7. By contrast, overrepresented GO terms for non-responsive genes included various kinds of housekeeping functions (e.g. "protein transport", "RNA processing", "translation", "cell cycle phase", and "DNA repair"). It is well known that housekeeping genes have stable expression levels in various kinds of cells and tissues [8,21], but our data shows that housekeeping genes are also somehow shielded from a wide variety of artificial disturbances. This finding indicates that genes with regulatory and housekeeping functions have clearly different dynamic states.

**Table 1:** Gene Ontology (GO) categories over-represented in groups of responsive and non-responsive genes

Group of genes	GO id	GO name	N genes	p-value	Enrichment ratio
Responsive genes with CpG islands	GO:0048513	Organ development	543	0	2.233
	GO:0003700	Transcription factor	323	0	2.198
	GO:0007399	Nervous system devel.	268	0	2.351
	GO:0007155	Cell adhesion	211	0	2.032
	GO:0006928	Cell motility	134	6.6027E-12	2.095
	GO:0009888	Tissue development	125	1.0458E-13	2.322
	GO:0031012	Extracellular matrix	115	3.245E-11	2.175
	GO:0001568	Blood vessel devel.	107	0	3.313
	GO:0007507	Heart development	85	2.8866E-15	3.122
	GO:0016055	Wnt receptor signaling	60	1.1637E-10	3.008

Non-responsive genes with CpG islands	GO:0015031	Protein transport	370	0	2.147
	GO:0006396	RNA processing	236	0	3.403
	GO:0006412	Translation	228	0	3.154
	GO:0044429	Mitochondrial part	206	4.4409E-16	2.475
	GO:0022403	Cell cycle phase	161	4.5759E-10	2.141
	GO:0016887	ATPase activity	158	1.3717E-10	2.278
	GO:0008380	RNA splicing	133	0	5.358
	GO:0006281	DNA repair	124	1.6187E-13	3.088
	GO:0006260	DNA replication	92	1.5896E-09	2.864
	GO:0006457	Protein folding	78	6.4245E-07	2.484

### TF-responsiveness of genes in ES cells is correlated with tissue-specific expression upon cell differentiation

**Figure 2:**
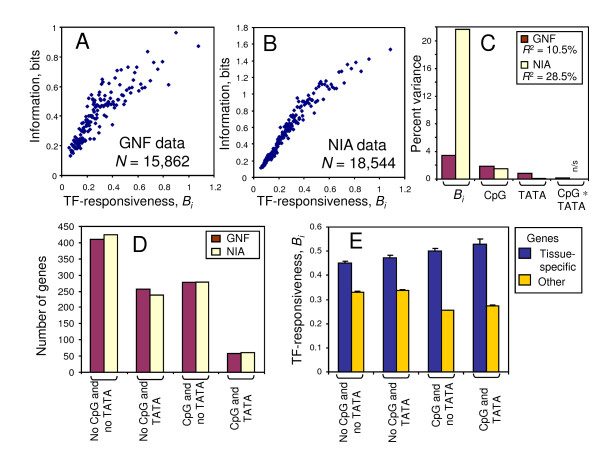
**Association of TF-responsiveness in ES cells with tissue-specificity**. (A, B) Scatterplot of average tissue-specificity measured by the information of mRNA abundance in: GNF and NIA databases, respectively, versus TF-responsiveness (*B*_*i*_); each dot represents the average for 100 genes with similar TF-responsiveness. (C) Percent unique variance of tissue-specificity (information) explained by TF-responsiveness (*B*_*i*_), presence of CpG island (CpG), presence of TATA box, and interaction of the two later factors (CpG*TATA), based on multi-variate linear regression; "n/s" = non-significant, otherwise it is significant (*p *< 0.05). (D) Fraction of the top 1000 tissue-specific genes that fall into four categories based on the presence of a CpG island (CpG) and TATA box. (E) Comparison of TF-responsiveness in tissue-specific genes from the GNF database (same as D) and in other genes, all differences are significant (*p *< 0.05).

Because non-responsiveness appeared to be associated with housekeeping functions, we decided to check if, on the contrary, genes that are responsive in ES cells are associated with tissue-specific functions in differentiated cells. We used two datasets on murine gene expression: "NIA Differentiated cells/tissues" and "GNF Mouse tissues" (Additional Files 8, 9). The degree of tissue specificity of genes was quantified by the information measure, which is based on Shannon's entropy [21]. We found that the information measure increased with increasing TF-responsiveness of genes in ES cells (Figure 2A, B). The results were consistent among two databases, but the relationship between TF-responsiveness and tissue specificity was stronger in the NIA database. The results thus indicate that responsive genes in undifferentiated ES cells tend to become tissue-specific upon differentiation.

It is conceivable, however, that the association of TF-responsiveness with tissue-specificity may have resulted from a non-random selection of TFs used in our perturbation experiments. To address this issue, we repeated the analysis after dividing the data to 3 functional subsets of TFs according to their expression in ES cells and differentiated organs and cells, as well as information measure of tissue specificity: (1) ES cell-specific (*Dnmt3b, Eed, Gadd45a, Id1, Klf4, Mybl2, Mycn, Nanog, Nr0b1, Nr5a2, Pou5f1, Sall4, Sox2, Tcea3, Whsc2, and Zscan4c*), (2) tissue-specific (*Ascl1, Ascl2, Cdx2, Dlx3, Eomes, Esx1, Gata3, Mef2c, Msc, Myod1, Otx2, Rhox6, Sfpi1, Sox9, and T*), and (3) widely expressed (*Aes, Atf3, Cbx8, Ctnnb1, Elf1, Etv3, Foxj2, Myc, Nr2f2, Nrip1, Rxra, Smad1, Smad4, Smad7, Stat3, Suz12, Tcf3, Tcf4, and Zfand3*). These subsets of induced TFs yielded highly correlated values of TF-responsiveness (*r *> 0.7) (Additional File 10, A-C), which were strongly associated with tissue specificity of responding genes for all subsets of manipulated TFs (Additional File 10, D-F). Thus, the association between TF-responsiveness with tissue-specificity is stable and does not seem to be related to the function of manipulated TFs.

**Figure 3:**
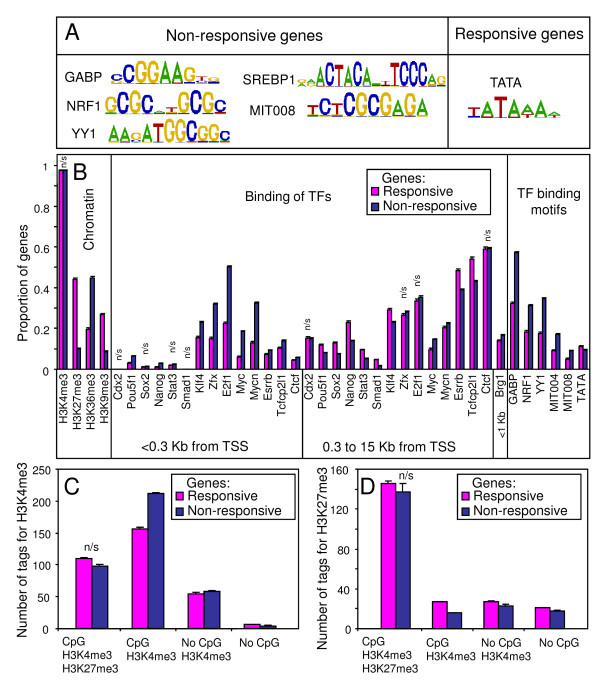
**Chromatin modifications, binding of transcription factors, and binding motifs in promoters of responsive and non-responsive genes**. (A) DNA binding motifs over-represented in the promoters (<200 bp from TSS) of non-responsive and responsive genes with CpG islands; (B) Comparison of chromatin modifications [7,18], binding of transcription factors [9,16,17], and binding motifs among responsive and non-responsive genes with CpG islands; "n/s" = non-significant, otherwise it is significant (*p *< 0.05); (C) Average strength of H3K4 tri-methylation measured by the number of ChIP-seq tags within 1 Kb from TSS of responsive and non-responsive genes, "n/s" = non-significant; (D) Average strength of H3K27 tri-methylation measured by the number of ChIP-seq tags within 3 Kb from TSS of responsive and non-responsive genes, "n/s" = non-significant. Numerical data for (C) and (D) is in Additional File 14.

It has been reported that tissue-specific genes tend to have a TATA box but no CpG islands [1,21]. Thus, we used linear regression to assess the effect of these factors, together with TF-responsiveness of genes in ES cells, on the degree of tissue-specificity. In both databases (GNF and NIA), the TF-responsiveness of genes in ES cells was the best predictor of tissue specificity (Figure 2C, Additional File 11). Considering that 67.5% of responsive genes have CpG islands, the correlation between TF-responsiveness and tissue-specificity may seem contradictory to the notion that tissue-specific genes have no CpG islands. This prompted us to further examine the characteristics of the top 1,000 tissue-specific genes selected on the basis of the highest information measure. We found that more than a third (33.4-34.1%) of these tissue-specific genes had CpG islands (Figure 2D). This is consistent with another observation that 24% of brain-specific promoters have CpG islands, although the proportion of tissue-specific promoters with CpG islands in other tissues is lower (9 - 14%) [22]. Our data showed that tissue-specific genes with CpG islands had higher TF-responsiveness in undifferentiated ES cells than non-tissue-specific genes with CpG islands (Figure 2E), indicating their special dynamic status. TATA box is over-represented among tissue-specific genes (30.1-31.4%) compared to other genes (15.1%), but it has only a weak association with TF-responsiveness of genes with CpG islands (Figure 3B).

### TF-responsiveness of genes in ES cells is correlated with histone modifications and binding of TFs

To examine the relationship between TF-responsiveness of genes and their known features, we analyzed available data on chromatin modifications and binding of various TFs to gene promoters in ES cells. First, we searched for possible overrepresented sequence motifs (defined *de novo*) in promoters of non-responsive and responsive genes using CisFinder [23] and identified GABP, NRF1, YY1, SREBP1, and MIT008 motifs for non-responsive genes (Figure 3A). Motif SREBP1 was described by [24] and MIT008 was over-represented in mammalian promoters [25], although the TF that binds to this motif remains unknown. The TATA box was over-represented in promoters of responsive genes with CpG islands. Next, we estimated the proportion of genes that carried specific histone modifications, binding of TFs based on published ChIP-seq data [7,16,18], and putative TF-binding motifs identified above. The most striking differences between non-responsive and responsive genes were observed for genes with CpG islands: responsive genes tended to bear the H3K27me3 and H3K9me3 chromatin marks, whereas non-responsive genes tended to have a H3K36me3 chromatin mark, binding of E2F1, ZFX, MYC, and MYCN within 300 bp from TSS, and binding motifs of GABP, NRF1, and YY1 in promoters (Figure 3B, Additional File 12). Among genes with no CpG islands, responsive genes were enriched in H3K4me3 chromatin marks and binding of several TFs to distal portions of promoters; however the effects of these factors were much weaker than for genes with CpG islands (Additional Files 12, 13).

In addition to qualitative categories of histone methylation, we examined quantitative "strength" of methylation as represented by the number of ChIP-seq tags within 1 Kb distance from TSS for H3K4me3 and 3 Kb distance from TSS for H3K27me3 based on data from [7]. Among genes with CpG islands, H3K4me3 chromatin, and no qualitatively-assigned H3K27me3 peaks, responsive genes had weaker H3K4 tri-methylation levels and stronger residual H3K27 tri-methylation levels than non-responsive genes (Figure 3C, D; Additional File 14).

**Figure 4:**
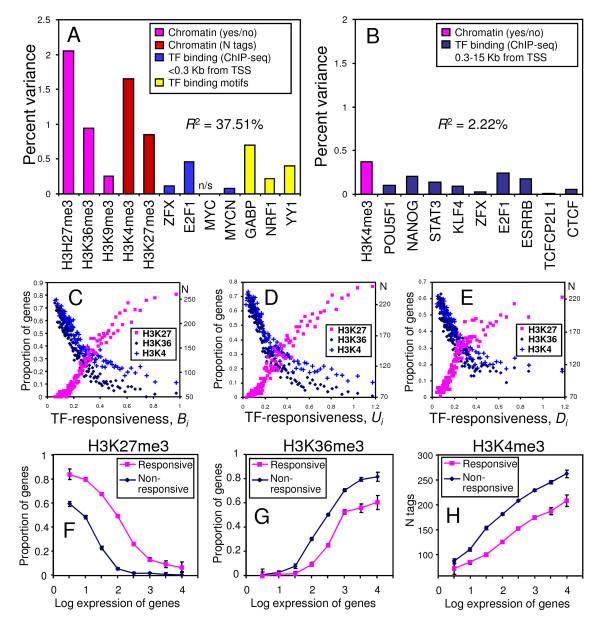
**Analysis of multiple factors associated with TF-responsiveness**. (A, B) Percent unique variance of TF-responsiveness (*B*_*i*_) explained by various factors based on multi-variate linear regression in the group of genes with CpG islands (A) and with no CpG islands (B); "n/s" = non-significant. (C, D, and E) Relationship between TF-responsiveness (*B*_*i*_, *U*_*i*_, and *D*_*i*_, respectively) among genes with CpG islands and the proportion of genes with H3K27me3 and H3K36me3 chromatin marks (scale on left side), and strength of H3K4 tri-methylation (number of ChIP-seq tags, scale on right side), estimated in groups of 100 genes with similar TF-responsiveness. (F, G, and H) Proportion of genes with H3K27me3 (F) and H3K36me3 (G) chromatin modifications, and strength of H3K4 tri-methylation (H) among responsive and non-responsive genes with CpG islands grouped by the level of their expression in ES cells; gene expression is estimated from RNA-seq data [19].

To further analyze the effect of major factors on the TF-responsiveness of genes, we used linear regression analysis, as this method helps distinguish true functional relations between cell characteristics from mere correlations [14]. Regression analysis of TF-responsiveness measured by the index *B*_*i *_(eq1) for the top 10 qualitative factors and 2 quantitative factors (histone methylation strength) identified from the comparison of responsive and non-responsive genes revealed that a large proportion of the variation in TF-responsiveness of genes with CpG islands could be attributed to chromatin modifications, binding of TFs, and the presence of TF binding motifs (*R*^2 ^= 37.51%) (Figure 4A, Additional File 15). Especially, the presence of H3K27me3 and H3K36me3 chromatin marks and the strength of H3K4 and H3K27 tri-methylation had a major unique contribution to the level of TF-responsiveness of genes. Binding of E2F1 and the presence of GABP binding motif also had a strong effect. By contrast, regression analysis of genes without CpG islands showed a weak dependency between TF-responsiveness and 10 top factors (*R*^2 ^= 2.22%) (Figure 4B, Additional File 15).

The strongest association of H3K27me3, H3K36me3, and H4K4me3 chromatin marks with the TF-responsiveness of genes with CpG islands prompted us to examine this relationship in greater detail. The proportion of genes with H3K27me3 marks, measured in groups of 100 genes with a similar level of TF-responsiveness, increased with increasing TF-responsiveness (*Bi*), whereas the proportion of genes with H3K36me3 marks and the strength of H3K4 tri-methylation decreased with increasing TF-responsiveness (*Bi*) (Figure 4C). This relationship remained the same even if TF-responsiveness of genes was measured by *Ui *or *Di *(Figure 4D,E), indicating that the association is not specific to either upregulated or downregulated genes. Similar relationships with chromatin marks were observed if responsiveness of genes was measured using the alternative data set "NIA Other Perturbations" (Additional File 16). Presence of the H3K27me3 chromatin marks among genes that were upregulated during differentiation of ES cells is consistent with the previous finding that these genes are silent in ES cells but poised for activation [7]. However, as this chromatin mark was also overrepresented among downregulated genes (Figure 4E), many genes with H3K27me3 marks were not fully suppressed in ES cells and were poised for both upregulation and downregulation. Strong downregulation of 5 genes with H3K27me3 chromatin marks, selected on the basis of microarray data, was previously confirmed by PCR [9]. Similarly, the presence of the H3K36me3 chromatin marks and strong tri-methylation of H3K4 among genes that were not upregulated following the induction of TFs is consistent with the notion that these genes are already active and thus cannot be activated further [7]. However, this reasoning does not seem to explain why genes with H3K36me3 chromatin and strong tri-methylation of H3K4 had low TF-responsiveness for downregulation (Figure 4D).

Because the association of H3K27me3, H3K36me3, and H3K4me3 chromatin marks with TF-responsiveness of genes can also be mediated by their effects on expression level, we analyzed the relationship between TF-responsiveness and chromatin status within groups of genes with similar expression levels. For simplicity, we limited the analysis to genes with CpG islands because they had a strong correlation between TF-responsiveness and chromatin status. The analysis revealed that the proportion of genes with H3K27me3 histone marks was consistently higher among responsive genes than among non-responsive genes with the same expression level (Figure 4F). By contrast, the proportion of genes with the H3K36me3 histone mark as well as the strength of H3K4 tri-methylation was lower among responsive genes than among non-responsive genes with the same expression level, except genes with very low expression, which had no H3K36me3 histone marks at all (Figure 4G,H). Taken together, the data indicate that association of H3K27me3, H3K36me3, and H3K4me3 chromatin marks with TF-responsiveness of genes is a novel dynamic feature of chromatin modifications and is not reduced to epigenetic control of stable gene expression levels.

## Discussion

This study provides the assessment of the dynamic status of mammalian genes in ES cells by the analysis of their TF-responsiveness to manipulation of 50 TFs and 3 other genes. Comparison with an independent data set shows that measurements of TF-responsiveness are reproducible. The group of responsive genes, which are readily upregulated or downregulated depending on the kind of perturbation, appears to be enriched in regulatory functions. The group of non-responsive genes with steady expression levels unchanged after various perturbations is enriched in housekeeping functions. Responsive genes in ES cells tend to become tissue-specific upon terminal differentiation. The TF-responsiveness of genes in ES cells appears to be the best predictor of tissue-specificity, which can be used in combination with other predictors (e.g., TATA box and CpG islands). Tissue-specific genes are enriched not only in the group of genes with a TATA box and no CpG island, as was found before [21], but also among genes with CpG islands that have high TF-responsiveness in ES cells. This is consistent with the previous estimate that 40% of genes with CpG islands show tissue restricted expression [26-28].

TF-responsiveness of genes with CpG islands has a strong association with chromatin modifications and binding of certain TFs to promoters. The proportion of genes with H3K27me3 chromatin marks increases, whereas the proportion of genes with H3K36me3 chromatin marks as well as the strength of H3K4 tri-methylation decreases with increasing TF-responsiveness of genes. It is well known that H3K27me3 marks suppress gene expression, and H3K36me3 marks are indicators of genes with high expression [7,29]. However, our finding shows that in addition to the effect on gene expression level, these chromatin modifications are associated with the TF-responsiveness of genes. Furthermore, we found that binding of several TFs (E2F1, ZFX, and MYCN) and the presence of TF binding motifs (NRF1, GABP, and YY1) in proximal regulatory regions are associated with low TF-responsiveness. Because these factors correlate negatively with the H3K27me3 chromatin mark [29], it is possible that they can control the type of chromatin modification (i.e., facilitate H3K36me3 and inhibit H3K27me3), and in this way indirectly reduce the TF-responsiveness of genes. However, linear regression shows that these TFs also have a direct negative effect on TF-responsiveness that is not mediated by chromatin modifications in ES cells.

How is gene TF-responsiveness formed and maintained in ES cells? Low TF-responsiveness can be caused by tightly closed chromatin, absence of TF binding sites, or missing cofactors of transcription regulation. Our finding that promoters of many low responsive genes are occupied by E2F1, ZFX, and MYCN, supports another possibility that they have a very stable transcription-initiation complex that occupies the promoter and prevents binding of other TFs. Alternatively, it is conceivable that low TF-responsiveness of genes can be maintained actively via a stabilizing effect of negative feedback: A slight increase of transcription caused by external perturbation could increase the methylation of H3K36 [30], which, in turn, increases deacetylation of histones and decreases the level of expression, closing the negative feedback loop. For example, the Eaf3 protein (subunit of the Rpd3S histone deacetylase complex) in yeast binds to H3K36me3 and H3K36me2, causing deacetylation of histones [31-33]. Although the main function of this effect is to suppress cryptic promoters in the coding region [31], it may also cause some decrease in the rate of normal transcription.

High TF-responsiveness of genes could be explained by the presence of TF binding sites in the promoter, de-condensed chromatin, and the presence of cofactors of transcriptional regulation. However, mechanisms that amplify the stimulating effect of TFs via positive feedback could also be conceived. For example, it is possible to consider H3K27me3 histone modification as a key player, as it is abundant among responsive genes with CpG islands and has the strongest association with TF-responsiveness (Figure 4A). Binding of TFs to promoters marked with H3K27me3 initiates the first round of transcription but subsequently removes the H3K27me3 histone mark because the elongating form of RNA polymerase II is known to associate with UTX demethylase [34]. As the repressive chromatin domain shrinks, the rate of transcription increases, causing further reduction of H3K27me3 marks.

Because this is the first study of dynamic status of gene expression, many questions remain un-answered. It would be interesting to quantify the TF-responsiveness of genes in differentiated cells and check if the same factors are associated with responsive and non-responsive genes. After elucidating the mechanisms of TF-responsiveness, we could create conditions where certain genes would be effectively activated following specific treatments, or maintain stable expression levels in fluctuating environments. Finally, an understanding of dynamic gene expression profiles can help to reconstruct transcription regulatory networks because potential main nodes of this network are limited to the set of responsive genes.

## Conclusions

Responsiveness of gene expression to perturbations is a new way to characterize the dynamic status of genes. Responsive genes mostly have regulatory functions and a tendency to become tissue-specific upon differentiation, whereas non-responsive genes are enriched in housekeeping functions. Responsive genes mostly have H3K27me3 chromatin marks at their promoters, and non-responsive genes are associated with H3K36me3 chromatin, stronger tri-methylation of H3K4, binding of E2F1, and GABP binding motifs in promoters.

## Methods

TF-responsiveness was measured from the published data on the change of gene expression following induced overexpression of 50 TFs and 3 other genes [9]. Expression of a transgene inserted in the ROSA26 locus was induced by doxycycline withdrawal (Dox-), whereas control cells were continuously cultured in a Dox+ condition. The effect of TF manipulation was measured by the logratio of gene expression (Dox-/Dox+) on the 2nd day after induction. We analyzed only those genes whose expression was determined with sufficient accuracy (so that 1.5-fold changes were statistically significant) and whose TSS were known. Thresholds of TF-responsiveness that separate responsive and non-responsive genes (Figure 1C) were estimated as 75-percentile and 50-percentile, respectively, for two groups of genes with log-expression (log10, RNA-seq) from 0.5 to 1.5 and from 2.5 to 3.5; then thresholds were linearly interpolated as a function of gene expression in ES cells.

The following organs from 30-week old male mice of C57BL/6 strain were used for gene expression profiling: brain cortex, cerebellum, eyes, skeletal muscle, heart, bone, liver, kidney, bladder, skin, visceral fat, lung, small intestine, large intestine, and stomach. Mouse husbandry and organ collection were approved by the Institutional Animal Care and Use Committee (ASP# 220-LG-2011). Although comparable data is available from the GNF database [15], the advantage of our data is that we used in-house designed microarrays[35] that represent a large set of genes (*N *= 25030), and probes for many genes are more sensitive compared to arrays used in the GNF database. Mice were euthanized by cervical dislocation. Total RNA was isolated by TRIzol (Invitrogen). Cy3-CTP labeled sample targets were prepared with total RNA by Low RNA Input Fluorescent Linear Amplification Kit (Agilent). Cy5-CTP labeled reference target was produced from mixture of Stratagene Universal Mouse Reference RNA and MC1 cells RNA. Samples were collected in 2 replications taken from different animals.

To characterize the effect of inhibitors, which are known to support the pluripotent state of ES cells [36], we treated B6R(5) mouse ES cells (C57BL/6 strain) with FGFR inhibitor PD173074 [37] (100 μM), MEK inhibitor PD98059 [38] (25 μM), and GSK-3 inhibitor BIO [39] (2 μM) 24 hr after plating. Cells were grown without feeders on gelatin-coated 6-well plates at 100,000 cells/well (10^4 ^cells/cm^2^) in complete ES medium, which was changed daily. Inhibitors dissolved in DMSO were added 24 hr after plating and cells were harvested 48 hr after treatment (72 hr after plating). Control cells were treated with DMSO. RNA was extracted and processed as described. Gene expression data were analyzed using the NIA Array Analysis [40].

Whole-genome data on chromatin modifications H3K4me3, H3K27me3, and H3K36me3 [7] were re-mapped to the latest mouse genome (mm9, NCBI/NIH) using the UCSC coordinate conversion tool (http://genome.ucsc.edu/cgi-bin/hgLiftOver). Tri-methylation of H3K4 and H3K27 was counted for a gene if methylation peaks identified using hidden Markov models and sliding windows was within 1 Kb from the TSS, whereas tri-methylation of H3K36 was counted along the entire transcript length. Genes with H3K9me3 marks with 5 Kb of TSS were taken from the reference [18]. Strength of H3K4me3 and H3K27me3 methylation was assessed by the number of ChIP-seq tags within 1 and 3 Kb from TSS, respectively, based on the data from the reference [7]. Expression levels were estimated using published RNA-seq data [19] and transcript coordinates from the NIA Mouse Gene Index, assembly mm9 [20], and expressed in log-transformed number of tags per 1 Kb transcript length. The RNA-seq method with random tags is better for comparing expression levels of different genes than microarrays because it does not have biases related to the position of the oligo and its sequence. Obtained gene expression values correlated well with microarray results (*r *= 0.71, Additional File 17). A few genes (*N *= 345) had no RNA-seq tags, possibly because tags may have been assigned to a different gene copy in the genome. The expression of these genes was interpolated from microarray data (i.e., gene expression in Dox+ conditions) using linear regression. To plot the relationship between TF-responsiveness and chromatin status, genes were ordered by increasing TF-responsiveness and split into sequential sets of 100 genes. We then estimated the average TF-responsiveness and the proportion of genes with a specific chromatin modification in each group of 100 genes.

The location of the main TSS for 17,412 non-redundant genes was taken from [7] and was identified using CisView [41] for 4,981 other genes. CpG islands were identified using CpGProD software [42] and attributed to genes if they were located within 1 Kb from the TSS. TF binding motifs over-represented in promoters of responsive and non-responsive genes were identified and annotated using CisFinder [23]. We analyzed genes with and without CpG islands separately, and for each group of genes we analyzed 200-bp regions upstream of the TSS and 200-bp regions downstream of the TSS (4 pairs of comparisons in total). Promoters of all genes (from -500 to +500 bp) were searched for the occurrence of TF binding motifs using CisFinder assuming 5 false positive matches per 10 Kb of random sequence. Thresholds for the matching score were further adjusted to a minimum of 2-fold enrichment of motif abundance either in the group of non-responsive genes or responsive genes. In particular, all motif matches were separated into groups according to their orientation ("+" or "-") and position relative to TSS in 100-bp intervals (i.e., from -500 to -400; from -400 to -300,..., and from 400 to 500). The combination of these two criteria yielded 20 groups of motif matches, which were analyzed separately. If the over-representation ratio of motif matches in a specific group was >2 fold, then all matches were counted in this group. However, if the over-representation ratio was <2 fold, then the matching threshold was increased to achieve the 2-fold enrichment. If the 2-fold enrichment was not achieved after any increase of the threshold, then no matches were counted in that group. Because TATA box has a strictly defined location in the promoter, it was handled separately from other TF binding motifs. TATA box was identified using the degenerative pattern KAWWW starting from 40 to 20 bp upstream of the TSS [41].

## Authors' contributions

AAS carried out the study design and the data analysis, and prepared figures and wrote the manuscript. AN participated in the study design, and carried out the experiments. YP carried out microarray experiments. LSC carried out RNA extraction from mouse organs. TA coordinated and performed mouse organ collection. MT and SM carried out the perturbations of ESCs by inhibitors of the FGFR, MEK, and GSK pathways. MSHK conceived of the study, and participated in its design, data analysis, and coordination, and wrote the manuscript. All authors read and approved the final manuscript.

## Supplementary Material

Additional file 1 **Data sets used in the study**. Click here for file

Additional file 2 **TF-responsiveness estimated from expression change in ES cells after induction of 50 transcription factors and 3 other genes **[9]. Click here for file

Additional file 3 **Data on chromatin status **[7], **binding of transcription factors **[9,16,17], **and presence of binding motifs in promoters of genes in mouse ES cells**. Click here for file

Additional file 4 **TF-responsiveness estimated from the "NIA Other Perturbations" data set**. Click here for file

Additional file 5 **Comparison of TF-responsiveness estimated from two databases: "NIA ES Bank, 53 genes", and "NIA Other Perturbations"**. Click here for file

Additional file 6 **Frequency distribution of mRNA decay rates (1/hr) **[14]**in groups of responsive and non-responsive genes**. Click here for file

Additional file 7 **Gene Ontology (GO) categories over-represented in groups of responsive and non-responsive genes**. Click here for file

Additional file 8 **Association between TF-responsiveness in ES cells and tissue specificity in the GNF database**. Click here for file

Additional file 9 **Association between TF-responsiveness in ES cells and tissue specificity in the NIA database**. Click here for file

Additional file 10 **Correlation of TF-responsiveness (Bi) estimated using three subsets of TFs: stem cell-specific, tissue-specific, and widely expressed**. (A-C) and their association with tissue-specificity, measured by information on the basis of gene expression in differentiated cells and tissues from NIA database (D-F). Each dot represents the average for 100 genes with similar TF-responsiveness. Click here for file

Additional file 11 **Predicting tissue specificity (information) with regression analysis**. Click here for file

Additional file 12 **Characterization of responsive and non-responsive genes.** Proportion of genes with chromatin modifications, binding of transcription factors (TFs), and TF binding motifs in promoters. Click here for file

Additional file 13 **Comparison of chromatin modifications **[7], **binding of transcription factors **[9,16,17], **and binding motifs among responsive and non-responsive genes with no CpG islands**. "n/s" = non-significant, otherwise significant (p < 0.05). Click here for file

Additional file 14 **Difference between responsive and non-responsive genes in the strength of H3K4 and H3K27 tri-methylation in promoters**. Click here for file

Additional file 15 **Regression analysis of the effect of various factors on the TF-responsiveness of genes**. Click here for file

Additional file 16 **Relationship between TF-responsiveness (Bi) measured from the alternative data set "NIA Other Perturbations" among genes with CpG islands and chromatin status**. Chromatin status is characterized by the proportion of genes with H3K27me3 and H3K36me3 chromatin marks (scale on left side), and strength of H3K4 tri-methylation (number of ChIP-seq tags, scale on right side), estimated in groups of 100 genes with similar TF-responsiveness. Click here for file

Additional file 17 **Correspondence between gene expression in mouse ES cells measured by microarrays **[9]**and RNA-seq methods **[19]. Click here for file
