# Supplementary material for: Responsiveness of genes to manipulation of transcription factors in ES cells is associated with histone modifications and tissue specificity
Source: BMC Genomics. 2011 Feb 9;12:102. doi: 10.1186/1471-2164-12-102 (PMC3044670; doi:10.1186/1471-2164-12-102)
Supplement: Additional file 5 — Comparison of TF-responsiveness estimated from two databases: "NIA ES Bank, 53 genes", and "NIA Other Perturbations". [file 1471-2164-12-102-S5.PPT]

## Slide 1
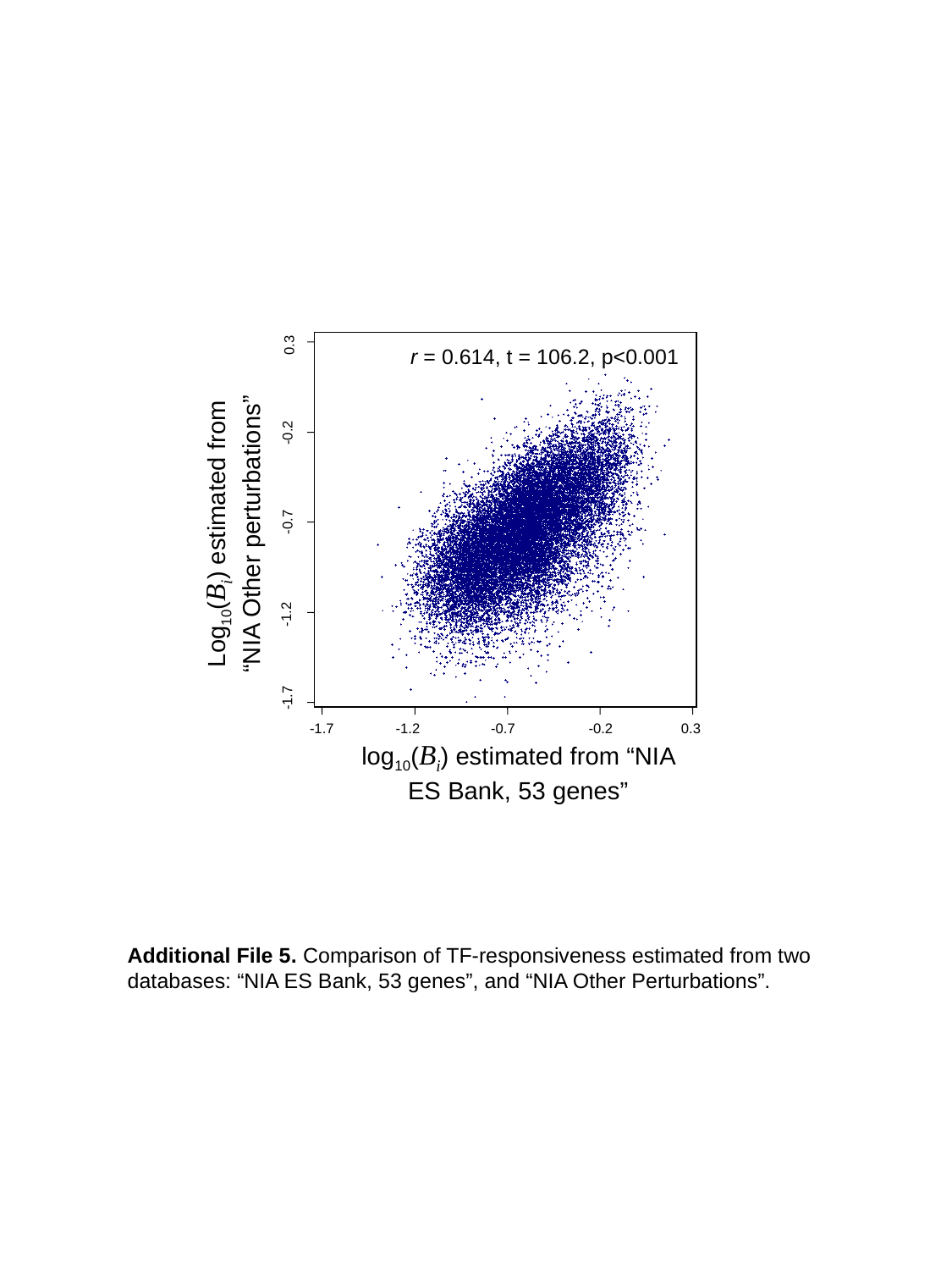

0.3
r = 0.614, t = 106.2, p<0.001
-0.2
Log10(Bi) estimated from “NIA Other perturbations”
-0.7
-1.2
-1.7
-1.7
-1.2
-0.7
-0.2
0.3
log10(Bi) estimated from “NIA ES Bank, 53 genes”
Additional File 5. Comparison of TF-responsiveness estimated from two databases: “NIA ES Bank, 53 genes”, and “NIA Other Perturbations”.
