# Supplementary material for: Responsiveness of genes to manipulation of transcription factors in ES cells is associated with histone modifications and tissue specificity
Source: BMC Genomics. 2011 Feb 9;12:102. doi: 10.1186/1471-2164-12-102 (PMC3044670; doi:10.1186/1471-2164-12-102)
Supplement: Additional file 6 — Frequency distribution of mRNA decay rates (1/hr) [14]in groups of responsive and non-responsive genes. [file 1471-2164-12-102-S6.PPT]

## Slide 1
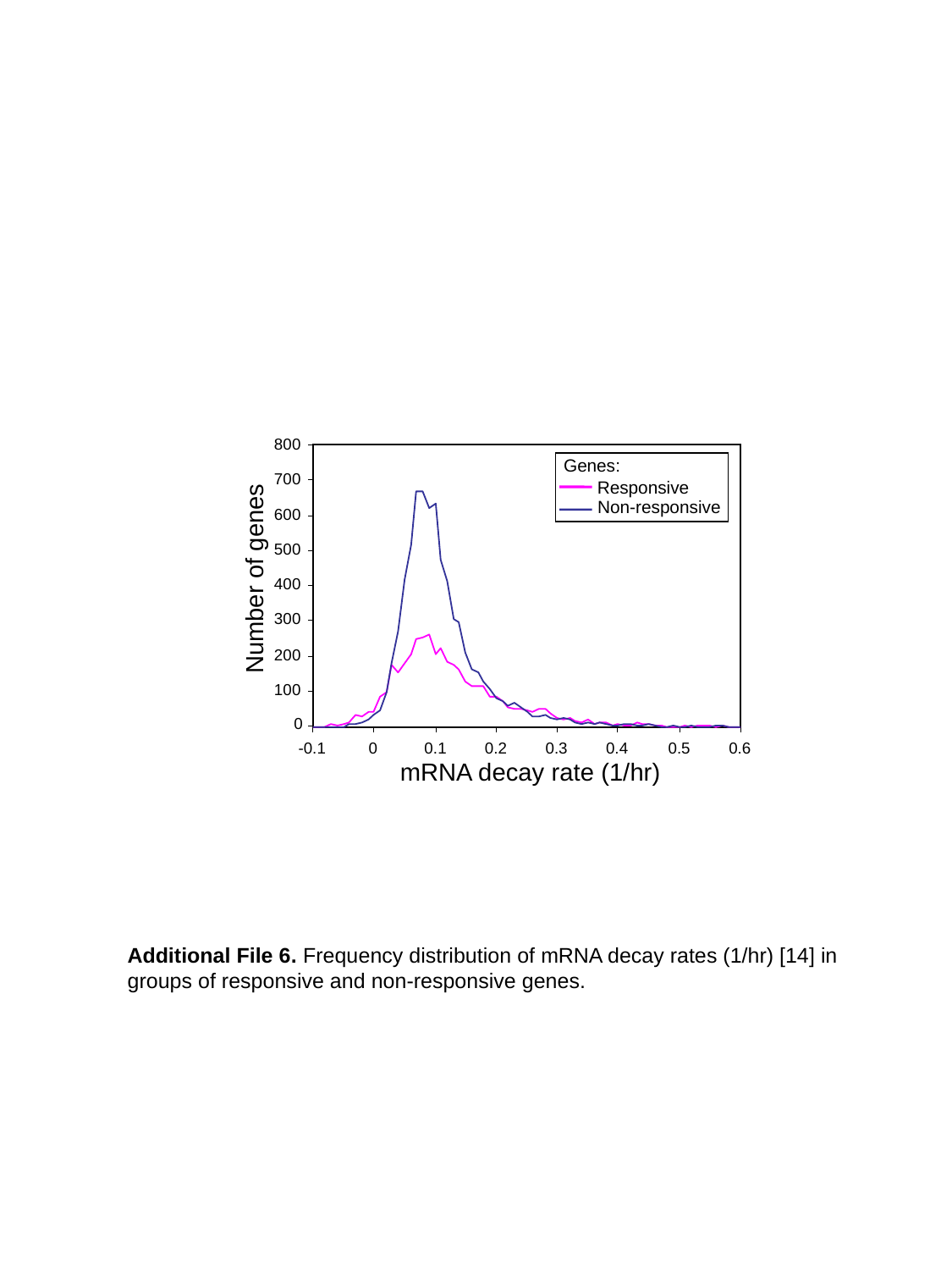

800
Genes:
Responsive
Non-responsive
700
600
500
Number of genes
400
300
200
100
0
-0.1
0
0.1
0.2
0.3
0.4
0.5
0.6
mRNA decay rate (1/hr)
Additional File 6. Frequency distribution of mRNA decay rates (1/hr) [14] in groups of responsive and non-responsive genes.
