# Supplementary material for: Responsiveness of genes to manipulation of transcription factors in ES cells is associated with histone modifications and tissue specificity
Source: BMC Genomics. 2011 Feb 9;12:102. doi: 10.1186/1471-2164-12-102 (PMC3044670; doi:10.1186/1471-2164-12-102)
Supplement: Additional file 10 — Correlation of TF-responsiveness (Bi) estimated using three subsets of TFs: stem cell-specific, tissue-specific, and widely expressed. (A-C) and their association with tissue-specificity, measured by information on the basis of gene expression in differentiated cells and tissues from NIA database (D-F). Each dot represents the average for 100 genes with similar TF-responsiveness. [file 1471-2164-12-102-S10.PPT]

## Slide 1
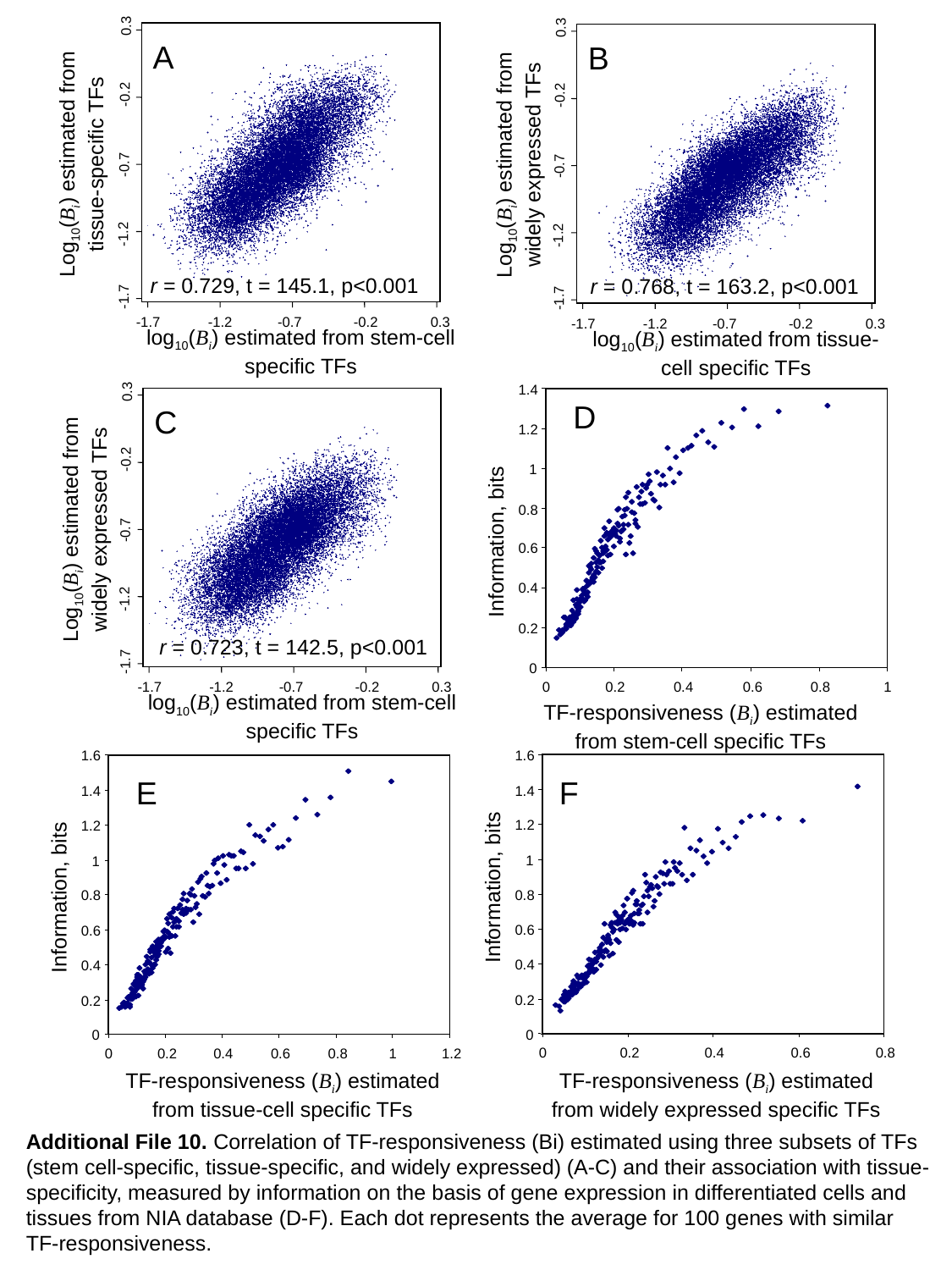

0.3
0.3
A
B
-0.2
-0.2
Log10(Bi) estimated from tissue-specific TFs
Log10(Bi) estimated from widely expressed TFs
-0.7
-0.7
-1.2
-1.2
r = 0.729, t = 145.1, p<0.001
r = 0.768, t = 163.2, p<0.001
-1.7
-1.7
-1.7
-1.2
-0.7
-0.2
0.3
-1.7
-1.2
-0.7
-0.2
0.3
log10(Bi) estimated from stem-cell specific TFs
log10(Bi) estimated from tissue-cell specific TFs
0.3
1.4
1.2
1
0.8
0.6
0.4
0.2
0
0
0.2
0.4
0.6
0.8
1
D
C
-0.2
Log10(Bi) estimated from widely expressed TFs
-0.7
Information, bits
-1.2
r = 0.723, t = 142.5, p<0.001
-1.7
-1.7
-1.2
-0.7
-0.2
0.3
log10(Bi) estimated from stem-cell specific TFs
TF-responsiveness (Bi) estimated from stem-cell specific TFs
1.6
1.4
1.2
1
0.8
0.6
0.4
0.2
0
0
0.2
0.4
0.6
0.8
1.6
1.4
1.2
1
0.8
0.6
0.4
0.2
0
0
0.2
0.4
0.6
0.8
1
1.2
E
F
Information, bits
Information, bits
TF-responsiveness (Bi) estimated from tissue-cell specific TFs
TF-responsiveness (Bi) estimated from widely expressed specific TFs
Additional File 10. Correlation of TF-responsiveness (Bi) estimated using three subsets of TFs (stem cell-specific, tissue-specific, and widely expressed) (A-C) and their association with tissue-specificity, measured by information on the basis of gene expression in differentiated cells and tissues from NIA database (D-F). Each dot represents the average for 100 genes with similar TF-responsiveness.
