# Supplementary material for: Responsiveness of genes to manipulation of transcription factors in ES cells is associated with histone modifications and tissue specificity
Source: BMC Genomics. 2011 Feb 9;12:102. doi: 10.1186/1471-2164-12-102 (PMC3044670; doi:10.1186/1471-2164-12-102)
Supplement: Additional file 13 — Comparison of chromatin modifications [7], binding of transcription factors [9,16,17], and binding motifs among responsive and non-responsive genes with no CpG islands. "n/s" = non-significant, otherwise significant (p < 0.05). [file 1471-2164-12-102-S13.PPT]

## Slide 1
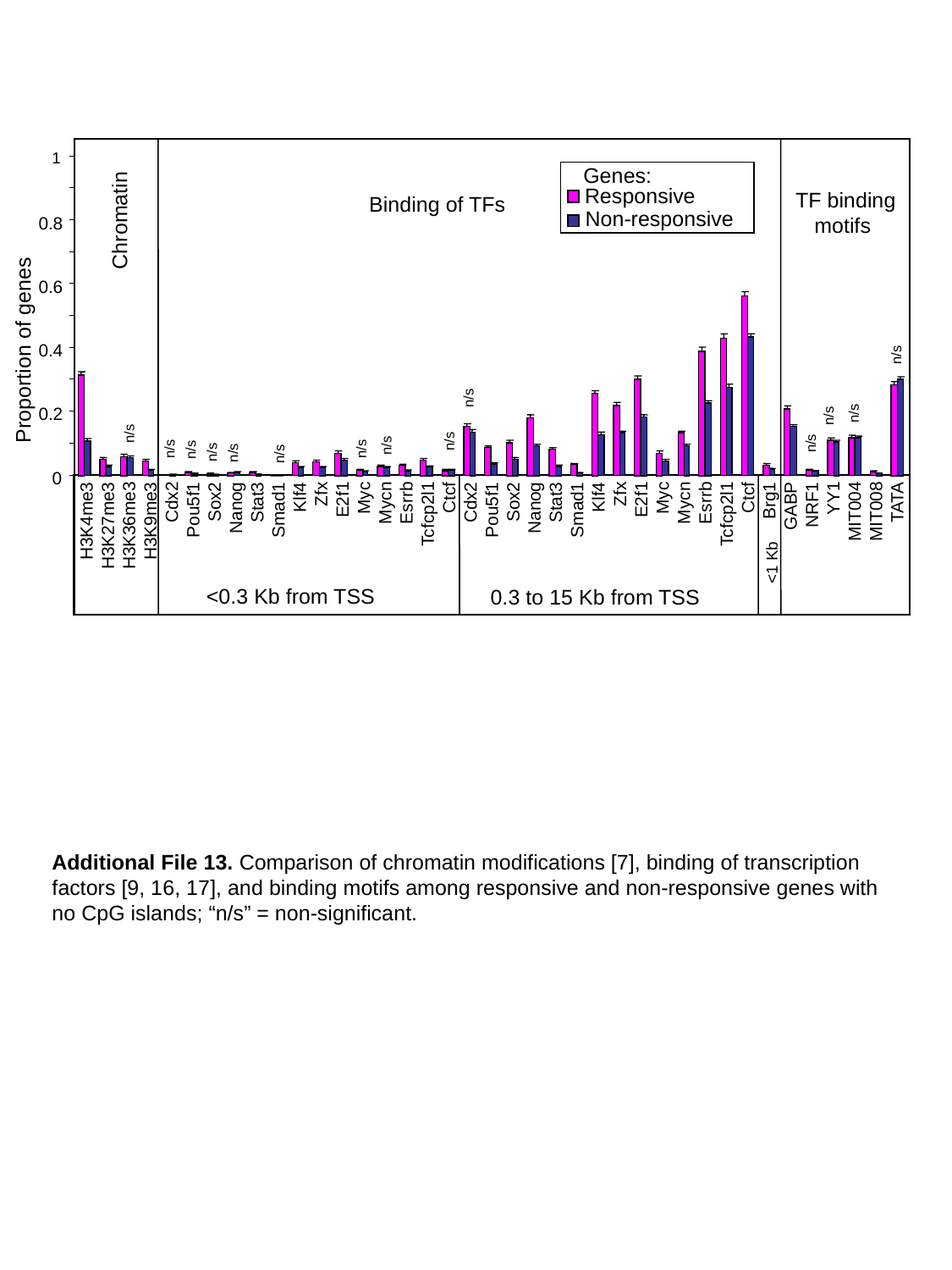

H3K4me3
H3K27me3
H3K36me3
H3K9me3
Cdx2
Pou5f1
Sox2
Nanog
Stat3
Smad1
Klf4
Zfx
E2f1
Myc
Mycn
Esrrb
Tcfcp2l1
Ctcf
Cdx2
Pou5f1
Sox2
Nanog
Stat3
Smad1
Klf4
Zfx
E2f1
Myc
Mycn
Esrrb
Tcfcp2l1
Ctcf
Brg1
GABP
NRF1
YY1
MIT004
MIT008
TATA
1
Genes:
Responsive
Non-responsive
TF binding motifs
Binding of TFs
Chromatin
0.8
0.6
Proportion of genes
0.4
n/s
n/s
n/s
n/s
0.2
n/s
n/s
n/s
n/s
n/s
n/s
n/s
n/s
n/s
n/s
0
<1 Kb
<0.3 Kb from TSS
0.3 to 15 Kb from TSS
Additional File 13. Comparison of chromatin modifications [7], binding of transcription factors [9, 16, 17], and binding motifs among responsive and non-responsive genes with no CpG islands; “n/s” = non-significant.
