# Supplementary material for: Responsiveness of genes to manipulation of transcription factors in ES cells is associated with histone modifications and tissue specificity
Source: BMC Genomics. 2011 Feb 9;12:102. doi: 10.1186/1471-2164-12-102 (PMC3044670; doi:10.1186/1471-2164-12-102)
Supplement: Additional file 16 — Relationship between TF-responsiveness (Bi) measured from the alternative data set "NIA Other Perturbations" among genes with CpG islands and chromatin status. Chromatin status is characterized by the proportion of genes with H3K27me3 and H3K36me3 chromatin marks (scale on left side), and strength of H3K4 tri-methylation (number of ChIP-seq tags, scale on right side), estimated in groups of 100 genes with similar TF-responsiveness. [file 1471-2164-12-102-S16.PPT]

## Slide 1
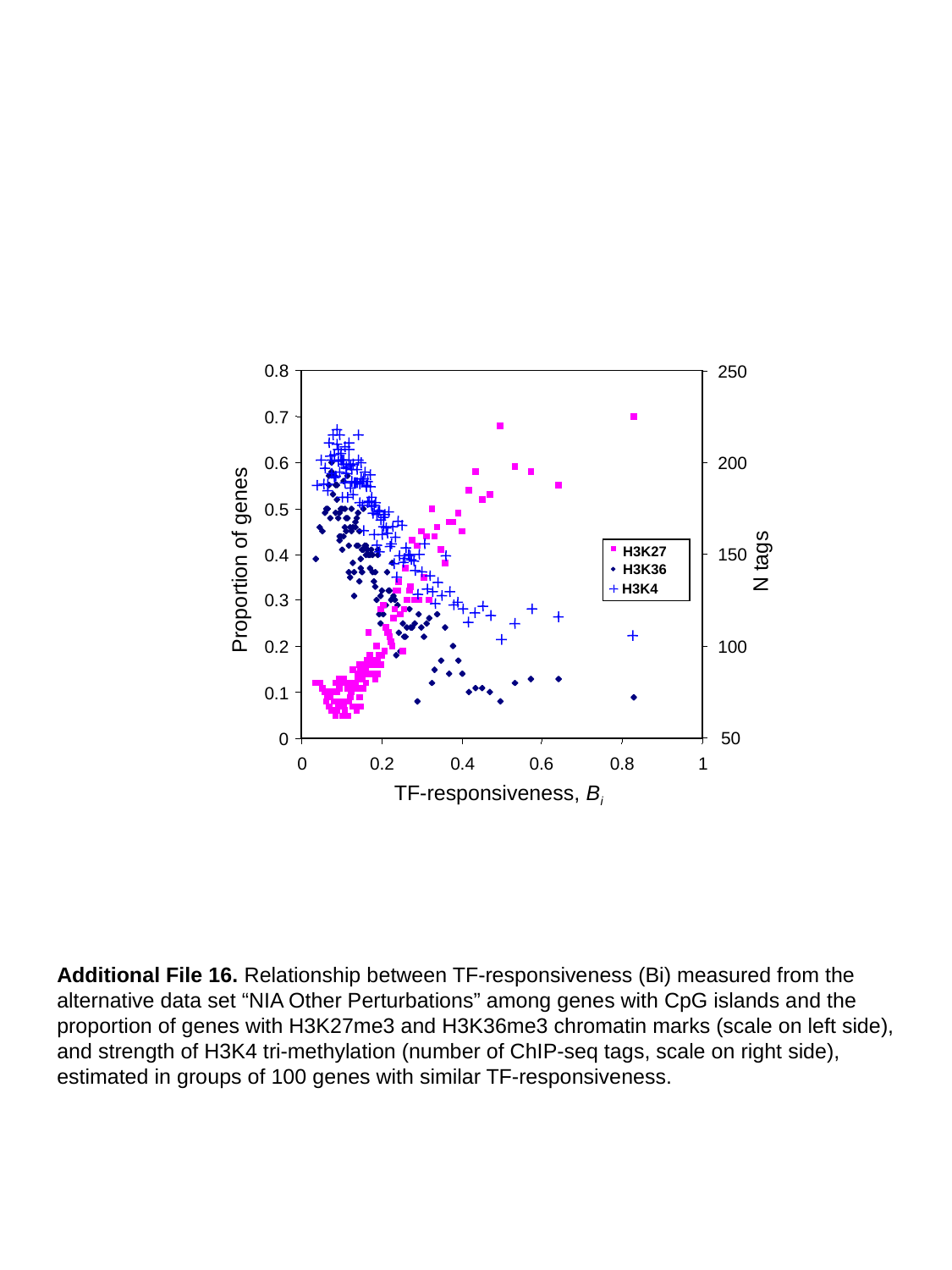

0.8
0.7
0.6
0.5
0.4
0.3
0.2
0.1
0
0
0.2
0.4
0.6
0.8
1
250
200
150
100
50
H3K27
H3K36
H3K4
Proportion of genes
N tags
TF-responsiveness, Bi
Additional File 16. Relationship between TF-responsiveness (Bi) measured from the alternative data set “NIA Other Perturbations” among genes with CpG islands and the proportion of genes with H3K27me3 and H3K36me3 chromatin marks (scale on left side), and strength of H3K4 tri-methylation (number of ChIP-seq tags, scale on right side), estimated in groups of 100 genes with similar TF-responsiveness.
