# Supplementary material for: Responsiveness of genes to manipulation of transcription factors in ES cells is associated with histone modifications and tissue specificity
Source: BMC Genomics. 2011 Feb 9;12:102. doi: 10.1186/1471-2164-12-102 (PMC3044670; doi:10.1186/1471-2164-12-102)
Supplement: Additional file 17 — Correspondence between gene expression in mouse ES cells measured by microarrays [9]and RNA-seq methods [19]. [file 1471-2164-12-102-S17.PPT]

## Slide 1
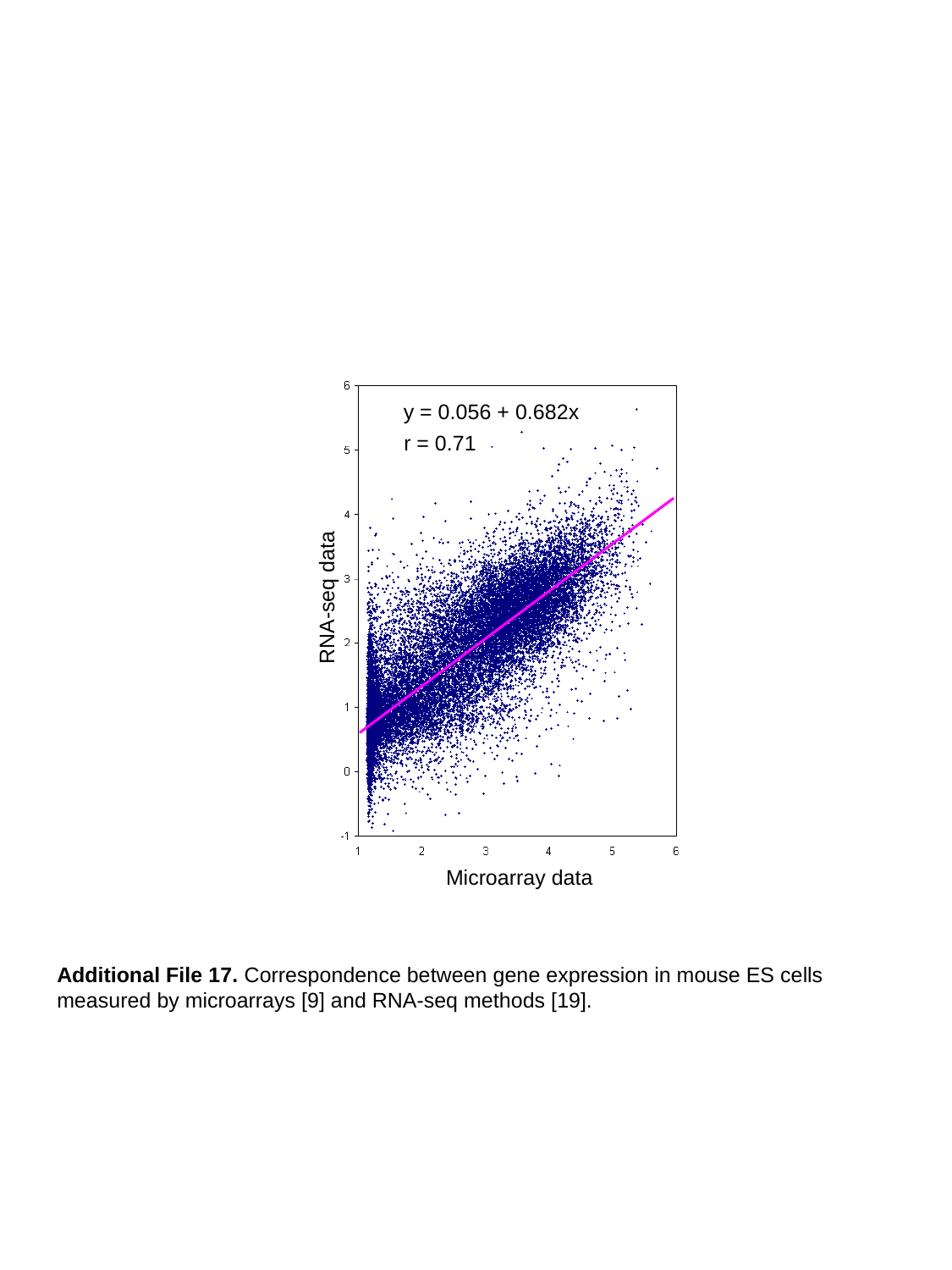

y = 0.056 + 0.682x
r = 0.71
RNA-seq data
Microarray data
Additional File 17. Correspondence between gene expression in mouse ES cells measured by microarrays [9] and RNA-seq methods [19].
